# Ankle Arthrodesis: Indications, Outcomes, and Patient Satisfaction

**DOI:** 10.7759/cureus.37177

**Published:** 2023-04-05

**Authors:** Sami Nogod, Alaa Mohamed Mohamed Khairy, Osama G Nubi, Montaser S Fatooh, Hozifa Mohammed Ali Abd-Elmaged

**Affiliations:** 1 Orthopaedics and Traumatology, Bashaer University Hospital, Khartoum, SDN; 2 Orthopaedic Surgery, Ribat University Hospital, Khartoum, SDN; 3 Orthopaedics and Traumatology, Al-Neelain University, Khartoum, SDN; 4 Orthopaedics and Traumatology, Alzaiem Alazhari University, Khartoum, SDN

**Keywords:** patient’s satisfaction, ankle joint arthrodesis, ankle arthrodesis, aofas, arthrodesis, osteoarthritis, ankle and foot, orthopaedic

## Abstract

Background

Osteoarthritis of the ankle, which has a prevalence of 3.4% in the general population and affects 70% of candidates post-traumatically, is less common than hip or knee arthritis. Despite the recent progress in ankle arthroplasty surgery, ankle arthrodesis is still considered the gold-standard treatment for severe osteoarthritis of the ankle joint and can achieve impressive improvements in pain and function in the majority of patients. Our objective is to assess the clinical and functional outcomes of ankle arthrodesis, postoperative complications, and patient satisfaction.

Methodology

This was a descriptive cross-sectional study that took place at Future Hospital in Khartoum, Sudan, from July 2015 to July 2022. Our institute performed 102 ankle fusion surgeries; 14 patients were excluded from the study because they did not show up for their follow-up visit, meaning 88 candidates were included. Two cannulated screws (6.5 mm) were used for ankle fixation in all patients. The outcome was assessed two years after surgery. The American Orthopaedic Foot and Ankle Society (AOFAS) score was used to assess ankle function before surgery and two years after the procedure. Postoperative complications and patient satisfaction were reported. The primary researcher collected data through direct interviews.

Result

The mean age of the study participants was 52.2 years, with 36.4% being between 40 and 50 years of age. The study consisted of 43 women and 45 men. The male-to-female ratio was almost 1:1. In 56.8% of candidates, the right ankle was involved. The overall rate of complications was 11.4%. Concerning patient satisfaction, 75% of the patients were fully satisfied. Moreover, significant correlations were found between the mean postoperative AOFAS score and age group, diabetes mellitus, complications, and patient satisfaction.

Conclusion

Ankle arthrodesis was demonstrated to be an excellent surgical method for reducing pain and enhancing ankle joint function. The most frequent complications were delayed wound healing, infection, and non-union. The functional outcome and patient satisfaction of this procedure were statistically high; patients with a higher functional score had higher levels of satisfaction. In contrast, diabetic and elderly patients had higher rates of complications.

## Introduction

Although total ankle arthroplasty procedures have seen remarkable progress recently, ankle fusion is considered the gold-standard treatment for severe osteoarthritis of the ankle. Ankle fusion achieves higher pain relief in the majority of patients [1,2]. However, it is known that additional degenerative changes can result from overloading the subtalar and Chopart joints, which may result in excruciating pain, additional surgeries, and a loss of function in the hindfoot. Several studies have evaluated the mid-term and long-term functional outcomes of ankle fusion [1-7], and others have compared the outcomes of ankle fusion and arthroplasty [8-11]. Good results were found in the majority of these cases, with high rates of patient satisfaction [1,5,12]. In their study, Ebalard et al. reported that 84% of patients complained of pain after a minimum follow-up of 10 years [13]. In other studies, the prevalence of osteoarthrosis ranged from 24% to 100% in the subtalar joint and from 18% to 77% in the Chopart joint [14]. It is currently unknown how frequently and how soon after an ankle fusion osteoarthrosis occurs in nearby joints. In this study, we did ankle fusion with a retrograde intramedullary nail without opening the ankle joint as in the standard procedure. When using the classical procedure of retrograde nailing for combined subtalar and ankle arthrodesis, several anatomical structures are at risk during dissection, and postoperative complications are increased, especially for those suffering from ulcers or other soft tissue problems. The progression of infection is high in patients with open procedures, especially when the ankle is involved. With percutaneous fusion, the exposure is minimal, the time of surgery is less, and the violation of soft tissues is avoided. Fuchs et al. did not find a correlation between the quality of life and the radiological grade of osteoarthrosis in the subtalar and Chopart joints [3]. The rate of non-union after ankle fusion is reported to range between 1% and 16% [6,7,12,15], with obesity and nicotine abuse being cited as risk factors for non-union in the current literature [16,17]. However, other studies do not fully support these findings. According to research by Ebalard et al., body mass index (BMI) and non-union rate are not correlated [13], whereas Collman et al. reported that obese patients are more likely to experience non-union [16]. This study aimed to know whether percutaneous fusion of the ankle joint in Charcot arthropathy patients has better clinical and functional outcomes and higher patient satisfaction than open procedures.

## Materials and methods

Methodology

Our study design was a descriptive cross-sectional study conducted at the Foot and Ankle Unit at Future Hospital, Khartoum, Sudan, while studying population and sampling, which involved 88 adult patients who underwent surgical fusion of the ankle joint at Future Hospital from July 2012 to July 2022.

Data collection and analysis

The primary researcher collected data after being trained to ensure the consistency and accuracy of the collected data. Through direct interviews, which included all the patients who underwent surgical fusion of the ankle joint at the time of the study. We excluded any patient with another foot deformity, patients who refused to take part in this study, and those who were lost to follow-up. Data were collected using a data collection sheet that included the patients’ demographics, complications, satisfaction, and American Orthopaedic Foot and Ankle Society (AOFAS) scores preoperative and postoperative, according to the first author. The AOFAS scoring system is a widely used tool for assessing ankle and foot function [18]. Each parameter in this score consists of nine questions covering three categories: alignment (out of 10 points), function (out of 50 points), and pain (out of 40 points). All three categories together equal 100 points. The data were cleaned and entered into a Microsoft Excel spreadsheet and analyzed using IBM Statistical Package for Social Sciences (SPSS) Statistics, version 28. Statistical t-tests were used to assess the demographic and functional score; a p-value less than 0.05 was considered significant.

Ethical considerations

Written consent was obtained from all patients for the procedure and their inclusion in this research, and the study was approved by the Research Ethics Review Committee at the Future Hospital, Khartoum, Sudan.

## Results

The study involved 88 patients who underwent surgical fusion of the ankle. The mean age was 52.6 years; 32% of candidates were in the 40-50 year age group, followed by 24% in the 50-60 year age group. The 88 participants comprised 45 male and 43 female patients. The right ankle was affected in 56.8% of cases, and the left side was affected in 43.2% of cases. In 86.4% of cases, a fracture was the cause of arthritis. The common comorbidities encountered were diabetes mellitus, rheumatoid arthritis, and poliomyelitis. Only three participants were smokers. The complication rate in all patients was 11.4%, with the most common complications being non-union (3.4%), wound dehiscence (3.4%), infection (2.3%), painful neuromas (1.1%), and metalwork failure (1.1%). Three patients who developed non-union required revisional surgeries, as shown in Table 1.

**Table 1:** The presentation of demographic characteristics, operation indications, and complications of patients who underwent ankle fusion surgery at Future Hospital (n=88)

		Frequency	Percentage (%)
Age group	< 40 years	7	8.0
	40–50 years	32	36.4
	51–60 years	24	27.3
	> 60 years	25	28.4
Gender	Female	43	48.9
	Male	45	51.1
Side	Left	38	43.2
	Right	50	56.8
Indications	Foot drop	3	3.4
	Idiopathic	2	2.3
	Post-traumatic	77	87.5
	Poliomyelitis	2	2.2
	Rheumatoid arthritis	4	4.5
Diabetes mellitus (DM)	No DM	76	86.4
	DM	12	13.6
Smoking	Yes	3	3.3
	No	85	96.6
Complication	Delayed wound healing	3	3.4
	Infection	2	2.3
	Metal failure	1	1.1
	Neuroma	1	1.1
	Non-union	3	3.4

Most patients were fully satisfied with the procedure (75%), some patients were fairly satisfied (20.5%), and a few patients were unsatisfied (4.5%). The mean preoperative AOFAS score was 35.89 (poor), which was improved to 85.71 (good), with a p-value of less than 0.001. Significant correlations were found between the functional outcome and the 40-50-year-old age group, male gender, idiopathic indication, risk factors (control of diabetes and mild smoking), delay in wound healing as complications, and full patient satisfaction, as illustrated in Table 2.

**Table 2:** Correlations between the postoperative AOFAS score and age, gender, ankle side, indications, smoking status, diabetes mellitus, complications, and patient satisfaction (n=88)

		Mean	Standard deviation	p-value
Age group	< 40 years	87.42	4.79	<0.001
	40–50 years	89.28	6.69	
	51–60 years	86.66	11.04	
	> 60 years	79.76	15.85	
Gender	Female	86.32	11.01	< 0.001
	Male	85.13	12.22	
Side	Left	85.84	12.53	< 0.001
	Right	85.62	10.95	
Indications	Foot drop	76.66	3.51	<0.001
	Idiopathic	93.00	2.82	
	OA (fracture)	86.24	12.01	
	Polio	75.50	2.12	
	Rheumatoid arthritis	83.75	5.73	
Smoking	Heavy	67.50	13.23	<0.001
	Mild	77.00	3.88	
	None	86.24	10.81	
Diabetes mellitus (DM)	Controlled DM	80.25	16.26	<0.001
	No DM	87.78	8.18	
	Uncontrolled DM	57.25	18.44	
Complications	Delayed wound healing	77.00	4.00	<0.001
	Infection	50.00	7.07	
	Metal failure	43.00	2.3	
	Neuroma	77.00	4.00	
	Non-union	55.33	18.77	
	None	88.79	5.95	
Satisfaction	Fair	76.66	8.87	<0.001
	Fully satisfied	90.54	4.26	
	Unsatisfied	46.75	5.56	

## Discussion

Ankle arthrodesis is used to improve the functional status of the ankle and foot as well as to decrease pain. This study focused on evaluating the functional outcome of patients following ankle fusion. We found that the mean age was 52.6 years, with a male-to-female ratio of almost 1:1. Post-traumatic arthritis was the most common type of arthritis found in this study (87.5%), which was comparable to the results of several other studies in the literature, such as those of Gaedk et al. (81%), Hendrickx et al. (87%), and Ebalard et al. [13].

The mean AOFAS score at two years was found to be 85.7; this is considered a good functional outcome but is expected to decrease with time as arthritis of the subtalar and Chopart joints evolves over time. Lower AOFAS scales were obtained by Gaedke et al. (62 in 9.6 years), Hendrickx et al. (67 in nine years), and Braito et al. (68.3 in 4.4 years). This discrepancy could be the result of the longer follow-up duration in these studies compared to that in our study. In our study, arthritis of the subtalar and Chopart joints was not prevalent because of the short follow-up duration. We found that 13.6% of our patients were diabetic, most of them had well-controlled diabetes, and 3.3% were smokers.

Postoperative complications were reported in 11.4% of candidates. Non-union and wound dehiscence were the most common complications (6.8%). Smokers had higher rates of non-union and significantly worse functional outcomes (i.e., a lower mean AOFAS score), as seen in Table 1. Diabetic patients with poorly controlled diabetes showed more frequent wound dehiscence than the other candidates, which is consistent with the findings of several other papers, such as those of Gaedk et al. [19].

Our study also elicited a major correlation between the mean AOFAS score and the complication rate, with higher scores among patients with no complications. Also, a significant correlation was found between age and functional outcome, with younger patients reporting higher levels of functional outcomes compared with older patients. Regarding patient satisfaction, this study found that most of the patients were fully satisfied (75%) with the procedure. The results also revealed a significant correlation between the mean AOFAS score and satisfaction, where patients with a higher AOFAS score had a higher level of satisfaction.

Limitations

The limitations of our study were a small sample size, a lack of participant diversity, resource scarcity, insufficient follow-up data availability, and socioeconomic circumstances.

## Conclusions

Despite the recent progress in ankle arthroplasty surgery, ankle arthrodesis is still considered the gold-standard treatment for severe osteoarthritis of the ankle joint and can achieve impressive improvements in pain and function in the majority of patients.

In our study, ankle fusion had excellent short-term and intermediate-term clinical and functional outcomes. Ankle arthrodesis was demonstrated to be an excellent surgical method for reducing pain and enhancing ankle joint function. The functional outcome and patient satisfaction of this procedure were impressive; patients with a higher AOFAS score had higher levels of satisfaction. The patients’ satisfaction levels were high overall and directly related to the functional outcome. Younger patients had better functional outcomes. Moreover, the pain reduction provided by this procedure was quite impressive. Smokers, diabetic patients, and elderly patients had higher complication rates, especially for wound dehiscence, infection, and non-union.
